# Protective Effects and Mechanisms of Pectolinarin against H_2_O_2_-Induced Oxidative Stress in SH-SY5Y Neuronal Cells

**DOI:** 10.3390/molecules28155826

**Published:** 2023-08-02

**Authors:** Qi Qi Pang, Ji Hyun Kim, Hyun Young Kim, Ji-Hyun Kim, Eun Ju Cho

**Affiliations:** 1 Department of Food Science and Nutrition & Kimchi Research Institute, Pusan National University, Busan 46241, Republic of Korea; pangqq@pusan.ac.kr; 2 Department of Food Science and Nutrition, Gyeongsang National University, Jinju 52725, Republic of Korea; jihyunkim@gnu.ac.kr (J.H.K.); hyunyoung.kim@gnu.ac.kr (H.Y.K.)

**Keywords:** pectolinarin, antioxidant, oxidative stress, hydrogen peroxide, SH-SY5Y neuronal cells, Alzheimer’s disease

## Abstract

This study aims to investigate the protective effects and mechanisms of pectolinarin against oxidative stress-induced cell damage in SH-SY5Y cells. Neurodegenerative diseases—such as Alzheimer’s disease—are potentially associated with oxidative stress, which causes excessive production of reactive oxygen species (ROS) that damage DNA and proteins in neuronal cells. The results of this study demonstrate that pectolinarin can scavenge hydroxyl and nitric oxide radicals in a concentration-dependent manner. Moreover, pectolinarin significantly increased cell viability while reducing ROS production and LDH release in the hydrogen peroxide (H_2_O_2_)-induced control group. Additionally, Pectolinarin recovered protein expression from H_2_O_2_-altered levels back to close-to-normal SH-SY5Y cell levels for components of the oxidative stress, inflammation, and apoptosis pathways—such as nuclear factor erythroid 2-related factor 2 (Nrf2), kelch-like ECH-associated protein (Keap1), anti-heme oxygenase 1 (HO-1), inducible nitric oxide synthase (iNOS), cyclooxygenase-2 (COX-2), interleukin-1β (IL-1β), B-cell lympho-ma-2 (Bcl-2) protein, and Bcl-2-associated X protein (Bax). These findings suggest that pectolinarin has the potential to be used as a plant material for functional foods to be applied in the treatment of neurodegenerative diseases, such as Alzheimer’s disease, by mitigating oxidative stress-induced damage to neuronal cells.

## 1. Introduction

The oxidative and antioxidant systems in human bodies are in dynamic equilibrium [1]. The imbalance between pro-oxidation and anti-oxidation overproduces reactive oxygen species (ROS), which induce oxidative stress [2]. ROS is a general term for substances that are more unstable than oxygen and mainly includes superoxide (O^2−^), hydroxyl (·OH), and hydrogen peroxide (H_2_O_2_) [3]. ROS combine with purines and pyrimidines in deoxyribonucleic acid (DNA), causing damage to the DNA double helix structure and causing oxidative stress, aging, and cancer [4,5]. Oxidative stress can also cause neuronal cell death and neuroinflammation in the brain, resulting in cognitive impairment and memory problems as well as neurodegenerative diseases, including Alzheimer’s disease (AD) and Parkinson’s disease [6,7].

With increases in the oxidative–redox system. it eventually loses its equilibrium—with age and the influence of irregular diets resulting in an excess of ROS production and oxidative stress in our bodies [8,9]. Besides this, neurodegenerative diseases such as AD have been shown to be oxidative stress-related diseases [10,11]. H_2_O_2_ stimulates astrocytes in the cortex and hippocampus, which significantly increases iNOS, and iNOS catalyzes nitric oxide (NO) production in the brain [12]. In addition, oxidative stress is also associated with the expression of Nrf2. Nrf2 is an antioxidant response regulator that is released from Keap1, translocates to the nucleus, activates protective gene transcription, and promotes their expression [13]. Studies have shown that inadequate Nrf2 expression has been found in the brains of human AD patients [14,15]. Therefore, the interaction of Keap1 and Nrf2 appears to be related to their antioxidant capacity, which protects neurons from oxidative stress [16].

In human cells, apoptosis is divided into internal pathways and external pathways, with the internal pathway undergoing molecular changes such as the activation of Bcl-2 and the inactivation of the pro-apoptotic effector Bax [17,18]. Apoptosis can help in the removal of damaged or dysfunctional brain cells, such as neurons that have accumulated toxic proteins [19]. This process is important for maintaining the overall health and function of the brain. However, excessive or abnormal apoptosis may contribute to the progression of neurodegenerative diseases [20]. Therefore, studying the apoptosis mechanisms of SH-SY5Y neuronal cells after treatment with the neurotoxic fragment Aβ_25–35_ is important.

SH-SY5Y cells, which are human-origin neuronal cells, are commonly used in the study of neurodegenerative diseases [21,22,23]. Many studies have reported the use of SH-SY5Y cells for studying oxidative stress-induced inflammation and apoptosis [24,25,26]. H_2_O_2_-induced oxidative stress in the SH-SY5Y cell model has been used to confirm the protective effects of active compounds [27,28]. Meanwhile, pectolinarin is a flavonoid that has been identified as an active compound in a number of plants, including *Cirsium japonicum* var. *ussuriense*, *Lippia rubella*, and *Cirsium nipponicum* (Maxim.) [29,30,31]. Anti-inflammatory, anti-apoptosis, anti-bacterial, and anti-tumor properties have been reported in pectolinarin [32,33,34]. Moreover, some previous studies have demonstrated the pharmacokinetic profile and intestinal absorption of pectolinarin to verify its physiochemical properties. According to Mehta and Dhapte [35], pectolinarin in rat plasma—following the oral administration of *Cirsium japonicum* DC. extract—reached the maximum possible concentration quickly. Chen et al. [36] reported that the absorption of pectolinarin in the jejunum was the highest among the parts of the small intestine, using the everted rat intestinal sac method. In this study, we investigated the ·OH and NO radical scavenging ability of pectolinarin in vitro and the mechanisms of pectolinarin against H_2_O_2_-induced oxidative stress in SH-SY5Y neuronal cells.

## 2. Results

### 2.1. Hydroxyl Radical (OH) Scavenging Activity of Pectolinarin

The results of the ·OH radical scavenging activity of pectolinarin are shown in Table 1. Pectolinarin (1, 2.5, 5, and 10 μg/mL) scavenged 63.09%, 80.43%, 83.60%, and 84.77% of the ·OH radicals, respectively. Furthermore, the scavenging activity at concentrations of 2.5, 5, and 10 μg/mL was greater than 80%. These results suggest that pectolinarin has antioxidant properties that make it capable of scavenging ·OH radicals.

### 2.2. NO Radical Scavenging Activity of Pectolinarin

The results for the NO radical scavenging activity of pectolinarin are shown in Table 2. Pectolinarin (1, 2.5, 5, and 10 μg/mL) showed dose-dependent increases in NO radical scavenging activity—at 23.21%, 24.50%, 30.40%, and 35.19%, respectively. These results suggest that pectolinarin possesses antioxidant properties that make it capable of scavenging NO radicals.

### 2.3. Effects of Pectolinarin against H_2_O_2_-Induced Cell Viability in SH-SY5Y Cells

**Figure 1:**
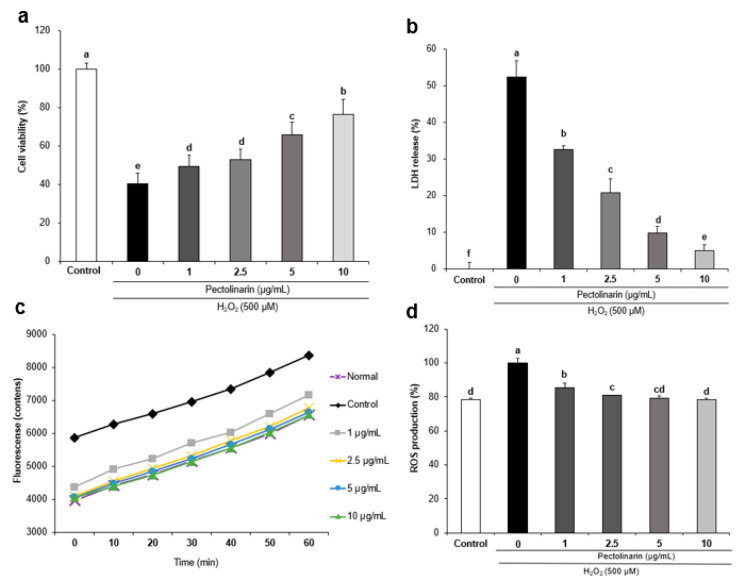
Protective effects of pectolinarin against H_2_O_2_-induced cell viability (**a**), LDH release (**b**), time course of change in intensity of DCF fluorescence during 60 min (**c**) and the intensity of ROS production at 60 min (**d**) in SH-SY5Y cells. Values are mean ± SD. The different letters (a–f) between groups represent significant differences as determined by the Tukey HSD multiple range test (*p* < 0.05).

The 3-(4,5-dimethyl-2-thiazolyl)-2,5-diphenyl-2H-tetrazolium bromide (MTT) assay was used to confirm the cell viability recovery effect of pectolinarin in SH-SY5Y cells after treatment with H_2_O_2_ (500 μM; Figure 1a). The cell viability of the control group was 40.26%, whereas the cell viability of the normal group was 100%—confirming that H_2_O_2_ was toxic to the SH-SY5Y cells. Pectolinarin (1, 2.5, 5, and 10 μg/mL) was used to treat the SH-SY5Y cells, and cell viability improved dependent on these increasing concentrations—reaching up to 49.20%, 52.72%, 65.82%, and 76.47%, respectively. These results show that pectolinarin protects SH-SY5Y cells from H_2_O_2_-induced cell toxicity.

### 2.4. Effects of Pectolinarin against H_2_O_2_-Induced Lactate Dehydrogenase Release in SH-SY5Y Cells

The effects of pectolinarin on H_2_O_2_-induced LDH release in SH-SY5Y cells are shown in Figure 1b. In comparison with the normal group—in which 0% of the LDH was released—in the control group, this significantly increased to 52.30%. Moreover, treatment with different concentrations (1, 2.5, 5, and 10 μg/mL) of pectolinarin in H_2_O_2_-induced SH-SY5Y cells dose-dependently decreased the level of LDH release to 32.59%, 20.83%, 9.87%, and 5.10%, respectively. These results show that pectolinarin inhibits H_2_O_2_-induced LDH release in SH-SY5Y cells.

### 2.5. Effects of Pectolinarin against H_2_O_2_-Induced ROS Formation in SH-SY5Y Cells

**Figure 2:**
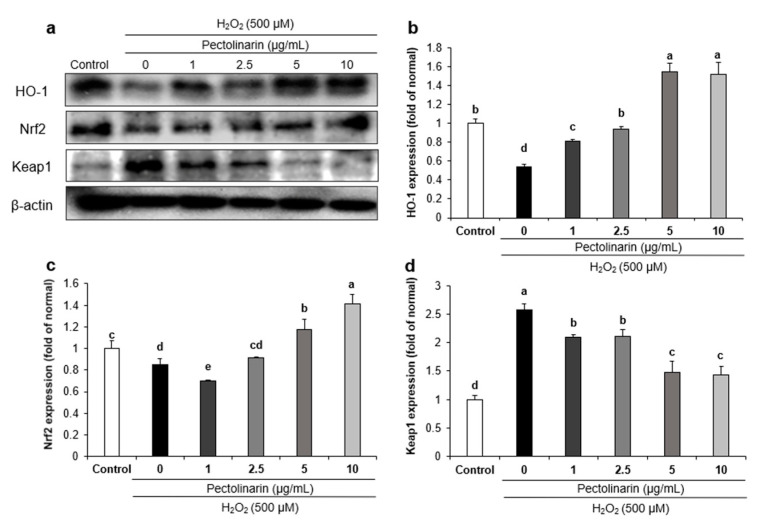
Effects of pectolinarin on oxidative stress-related protein expression in H_2_O_2_-induced SH-SY5Y cells. (**a**) Bands of HO-1, Nrf2, and Keap1 proteins expression level. (**b**) HO-1 protein expression level. (**c**) Nrf2 protein expression level. (**d**) Keap1 protein expression level. Values are mean ± SD. The different letters (a–e) among groups represent significant differences as determined by the Tukey HSD multiple range test (*p* < 0.05).

The dichlorofluorescein diacetate (DCF-DA) assay was used to confirm the inhibitory effects of pectolinarin against H_2_O_2_-induced ROS formation, and the results are presented in Figure 2. ROS production was significantly higher in the H_2_O_2_-induced control group (Figure 1c) when compared with the normal group, confirming that H_2_O_2_ caused oxidative stress in SH-SY5Y cells. As shown in Figure 2b, when the ROS production in the control group was 100%, the ROS production in the normal group was 78.28%—indicating that when the SH-SY5Y cells were exposed to H_2_O_2_ (500 μM) for 24 h and then reacted with DCF-DA, the ROS production in the control group increased by 21.72% compared with the normal group. The intensity of ROS production at 60 min (Figure 1d) showed that treatment with different concentrations (1, 2.5, 5, and 10 μg/mL) of pectolinarin dose-dependently decreased the levels of ROS to 85.65%, 80.92%, 79.32%, and 78.37% when the ROS production of the control group was set as 100%. These results show that pectolinarin inhibits H_2_O_2_-induced ROS formation in SH-SY5Y cells.

### 2.6. Effects of Pectolinarin on Oxidative Stress-Related Protein Expression in H_2_O_2_-Induced SH-SY5Y Cells

The action mechanisms of oxidative stress-related proteins such as HO-1, Nrf2, and Keap1 were measured using a Western blot to investigate the protective effects of pectolinarin against H_2_O_2_-induced oxidative stress in neural cells. The protein expression of HO-1 and Nrf2 was significantly lower in the H_2_O_2_-induced control group compared with the normal group, as shown in Figure 2. However, treatment with pectolinarin (1, 2.5, 5, and 10 μg/mL) in H_2_O_2_-induced SH-SY5Y cells increased Nrf2 and HO-1 protein expression. In addition, Keap1 protein expression was significantly higher in the H_2_O_2_-induced control group than in the normal group. On the other hand, treatment with pectolinarin (1, 2.5, 5, and 10 μg/mL) dramatically reduced Keap1 protein expression. These results suggested that pectolinarin has antioxidant properties via the regulation of oxidative stress pathway proteins in H_2_O_2_-induced SH-SY5Y cells.

### 2.7. Effects of Pectolinarin on Inflammation-Related Protein Expression in H_2_O_2_-Induced SH-SY5Y Cells

**Figure 3:**
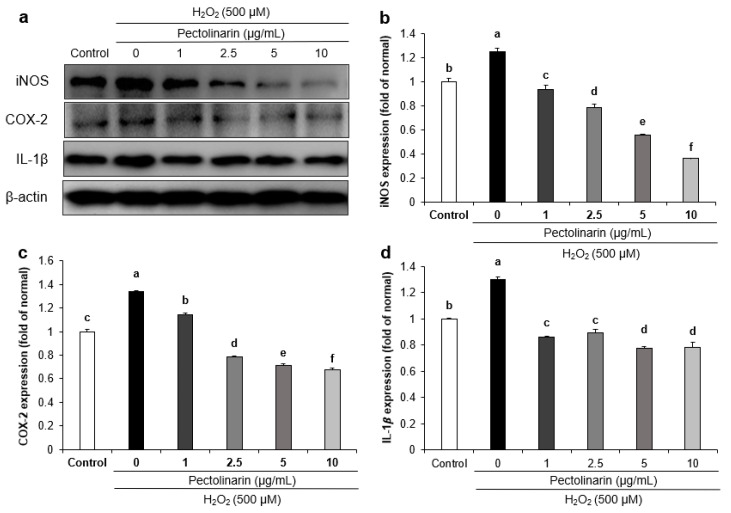
Effects of pectolinarin on inflammation-related protein expression in H_2_O_2_-induced SH-SY5Y cells. (**a**) Bands of iNOS, COX-2, and IL-1β proteins expression level. (**b**) iNOS protein expression level. (**c**) COX-2 protein expression level. (**d**) IL-1β protein expression level. Values are mean ± SD. The different letters (a–f) between groups represent significant differences as determined by the Tukey HSD multiple range test (*p* < 0.05).

Inflammation-related protein expression in H_2_O_2_-induced SH-SY5Y cells was assessed to confirm the mechanisms of pectolinarin. Figure 3 showed that H_2_O_2_-induced cells had significantly increased expression of IL-1β, iNOS, and COX-2 proteins compared with the untreated normal SH-SY5Y cells. Furthermore, pectolinarin (1, 2.5, 5, and 10 μg/mL) significantly reduced the expression of the iNOS, COX-2, and IL-1β proteins. These results show that pectolinarin inhibits inflammation in H_2_O_2_-induced SH-SY5Y cells by downregulating the inflammation pathway-related proteins iNOS, COX-2, and IL-1β.

### 2.8. Effects of Pectolinarin on Apoptosis-Related Protein Expression in H_2_O_2_-Induced SH-SY5Y Cells

**Figure 4:**
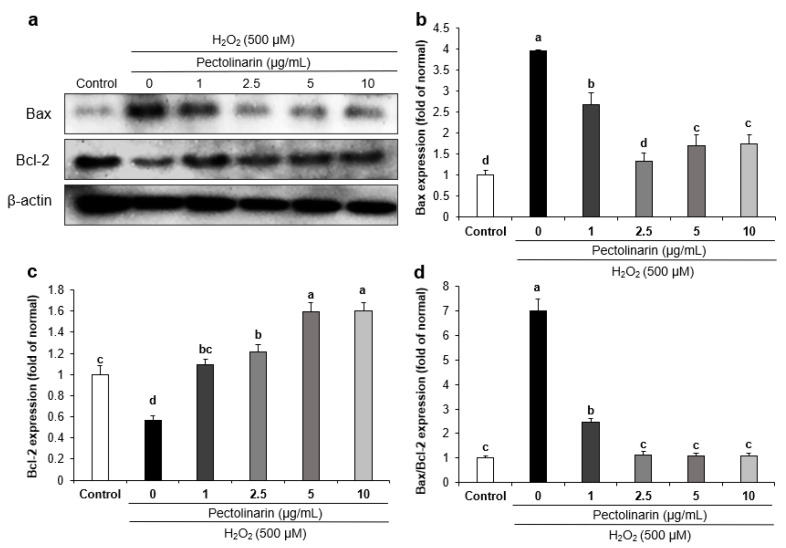
The effects of pectolinarin on apoptosis-related protein expression in H_2_O_2_-induced SH-SY5Y cells. (**a**) Bands of Bax and Bcl-2 proteins expression level. (**b**) Bax protein expression level. (**c**) Bcl-2 protein expression level. (**d**) Ratio of Bax/Bcl-2 proteins expression level. Values are mean ± SD. The different letters (a–d) between groups represent significant differences as determined by the Tukey HSD multiple range test (*p* < 0.05).

The mechanisms of apoptosis-related proteins were studied using Bax and Bcl-2 protein expression. As shown in Figure 4, H_2_O_2_-induced SH-SY5Y cells had significantly higher Bax protein expression than normal SH-SY5Y cells, but treatment with pectolinarin (1, 2.5, 5, and 10 μg/mL) significantly decreased the protein expression of Bax. On the other hand, treatment with H_2_O_2_ decreased Bcl-2 protein expression in SH-SY5Y cells compared with normal cells, and treatment with pectolinarin (1, 2.5, 5, and 10 μg/mL) significantly increased the protein expression of Bcl-2. Furthermore, the Bax/Bcl-2 protein expression was significantly higher in the H_2_O_2_-induced control group. These findings show that treatment with pectolinarin protects SH-SY5Y cells from H_2_O_2_-induced damage by modulating the expression of Bax and Bcl-2, which are proteins associated with apoptosis. 

## 3. Discussion

Under physiological conditions, the production and clearance of ROS in organisms are normal physiological functions [37]. Excessive ROS production in the body can readily lead to oxidative stress [38]. ROS consists of free radicals such as ·OH, O2− and other non-radicals such as H_2_O_2_ [39]. Although H_2_O_2_ is a toxic by-product, it is involved in many physiological processes such as cell proliferation, differentiation, and immune regulation [40]. However, high concentrations of H_2_O_2_ can promote cell apoptosis and oxidative stress in the brain, leading to neurodegenerative diseases such as AD [41,42].

The OH radical, as a by-product of ROS, has high reactivity and a strong oxidation capacity, which can damage cells or tissues in a short period of time [43]. Excess NO production in the brain can cause neuroinflammation and neurodegenerative diseases [44]. The present results showed that the ·OH and NO radical scavenging activity of pectolinarin increased dependent on its concentration, with its ·OH scavenging activity exceeding 80% at concentrations of 2.5, 5, and 10 μg/mL. These results suggest that pectolinarin has antioxidant properties for the scavenging of ·OH and NO radicals.

Several studies have found that throughout the natural aging process, various food intakes or physiological activities cause an increase in the excessive production of ROS, which promotes the deposition of Aβ and accelerates the formation of senile plaques [45,46]. The MTT assay was used in our present study to confirm the protective effects of pectolinarin against H_2_O_2_-induced oxidative stress in SH-SY5Y cells. The MTT assay is a standard approach for evaluating mitochondrial damage by measuring cell viability, and importantly, is well-known for assessing cell metabolism—in contrast with the sulforhodamine B assay, which assesses the protein contents [47]. The DCF-DA assay was used to confirm the inhibitory effects of pectolinarin against H_2_O_2_-induced ROS formation in SH-SY5Y cells. Dichlorofluorescein (DCF) is a highly fluorescent chemical formed by the oxidation of 2′,7′-dichlorodihydrofluorescein (DCFH) by ROS [48]. Meanwhile, we used the LDH cytotoxicity kit to confirm any inhibitory effects on LDH release. After being treated with pectolinarin, the SH-SY5Y cells showed an antioxidant effect by increasing cell viability and reducing ROS production. Besides this, treatment with pectolinarin lowered LDH secretion. These results suggest that pectolinarin can protect against H_2_O_2_-induced cell death, ROS production, and LDH increase.

Nrf2 is a transcription factor that regulates gene expression in antioxidant defense, detoxification, and anti-inflammatory pathways [49]. HO-1 is an enzyme that catalyzes heme breakdown to produce biliverdin, carbon monoxide, and iron. Biliverdin is then metabolized further to bilirubin, which has powerful antioxidant and anti-inflammatory properties [50]. Under normal conditions, Keap1 binds to Nrf2 and regulates Nrf2 activity by promoting ubiquitin-mediated degradation [51]. Studies have shown that activation of the Nrf2/HO-1 system can protect neurons against oxidative stress and prevent the development of neurodegenerative diseases [52,53]. In our present results, treatment with pectolinarin (1, 2.5, 5, and 10 μg/mL) in H_2_O_2_-induced SH-SY5Y cells showed a significantly increase in HO-1 and Nrf2 protein expression. In addition, treatment with pectolinarin (1, 2.5, 5, and 10 μg/mL) significantly decreased the expression of Keap1 protein. AD is closely related to the overproduction of ROS and oxidative damage in the brain, and research has shown that regulating the Nrf2/HO-1 pathway can also reduce ROS production, which is consistent with the results of this experiment [54]. These results suggest that pectolinarin has neuroprotective effects against oxidative stress induced by H_2_O_2_ through the regulation of antioxidant-related proteins in SH-SY5Y cells.

Inflammation is an immunological response to oxidative stress caused by an excess of ROS. NO is produced by the deamination of arginine under the action of iNOS and enters the adjacent cell through the cell membrane to cause inflammation [55]. COX-2 is an enzyme involved in the production of prostaglandins, as well as the promotion of inflammation and oxidative stress. Inhibiting COX-2 expression may have anti-aging effects by reducing inflammation and oxidative stress [56]. IL-1β is a biologically active small molecule protein that engages in cell signal transmission during the inflammatory response and produces additional pro-inflammatory cytokines [57]. This study found that H_2_O_2_-induced SH-SY5Y cells exhibited considerably higher levels of iNOS, COX-2, and IL-1β expression than H_2_O_2_-untreated SH-SY5Y cells. Furthermore, treatment with pectolinarin significantly decreased the expression of iNOS, COX-2, and IL-1β proteins. During the pathogenesis of AD, the inflammatory response causes neuronal damage and cell death. These proteins are involved in cell signaling in this pathogenesis process, which promotes the inflammatory response [58]; therefore, iNOS, COX-2, and IL-1β may be key players in the etiology of AD. These results suggest that pectolinarin protects H_2_O_2_-induced SH-SY5Y cells through regulating inflammation-related proteins.

Apoptosis is an active process that is regulated by a variety of genes [59]. H_2_O_2_ is the source of ·OH that can cross the cell membrane of the brain and cause DNA injury, lipid peroxidation, and oxidative stress—ultimately accelerating apoptosis in the brain [60]. Previous research has investigated apoptosis induction using various methods, such as by TUNEL assay, flow cytometry, and Western blots. Zhao et al. [61] confirmed that H_2_O_2_-treatment significantly induced apoptosis in SH-SY5Y cells using the TUNEL assay and flow cytometry with an Annexin V-FITC/PI kit. Moreover, Tian et al. [62] reported that H_2_O_2_-induced SH-SY5Y cells increased the expression ratio of Bax/Bcl-2, which is a valuable predictor of apoptosis. In the present study, the effect of pectolinarin on H_2_O_2_-induced apoptosis was studied using Western blots for the Bax/Bcl-2 ratio. The Bcl-2 protein family is classified into two categories: the pro-apoptotic protein Bax and the anti-apoptotic protein Bcl-2. The main mechanism of these two proteins is that Bax improves the permeability of the mitochondrial membrane and promotes cytochrome C protein release, ultimately leading to neuronal cell death [63,64]. On the other hand, Bcl-2 protects neuronal cells against death, apoptosis, and oxidative stress stimulation [65]. Our study showed that Bax/Bcl-2 protein expression was significantly increased in the H_2_O_2_-induced control group, but treatment with pectolinarin significantly reduced the Bax/Bcl-2 protein expression. These results suggest that pectolinarin protects H_2_O_2_-induced SH-SY5Y cells by regulating apoptotic pathway proteins. These results show that pectolinarin could regulate the expression of Bax and Bcl-2 proteins, reducing H_2_O_2_-induced oxidative stress and apoptosis in SH-SY5Y cells. This suggests that pectolinarin, as a potential natural compound in functional foods for use against AD, exhibits anti-oxidative stress and cytoprotective effects.

## 4. Materials and Methods

### 4.1. Reagents

**Figure 5:**
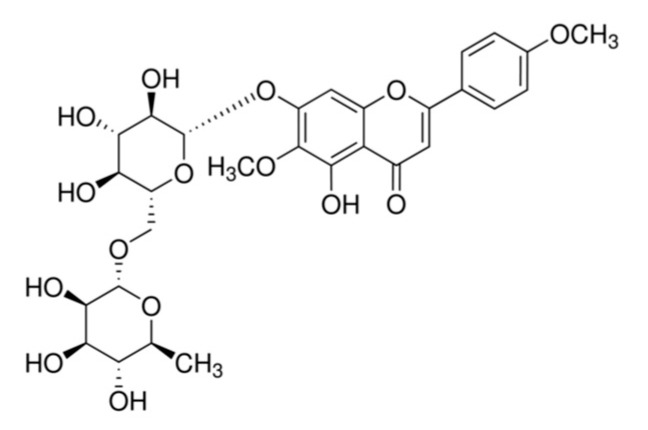
Structure of pectolinarin.

Thiobarbituric acid (TBA) was acquired from Lancaster Synthesis (Ward Hill, MA, USA), and trichloroacetic acid (TCA) was acquired from Kanto Chemical Co., Inc. (Tokyo, Japan). Dulbecco’s modified eagle medium (DMEM), fetal bovine serum (FBS), penicillin-streptomycin, and trypsin–ethylenediaminetetraacetic acid (EDTA) solution were obtained from Welgene (Daegu, Korea). The LDH cytotoxicity detection kit was purchased from Takara Bio (Siga, Japan). Dimethyl sulfoxide (DMSO) was purchased from Daejung (Siheung-si, Gyeonggi-do, Korea). The MTT was purchased from Bio Pure (Kitchener, ON, Canada). Pectolinarin and DCF-DA were purchased from Sigma-Aldrich (St. Louis, MO, USA). Sodium pentacyanonitrosylferrate (III) dihydrate (SNP) and H_2_O_2_ were purchased from Junsei (Tokyo, Japan). The protease inhibitor cocktail was obtained from Calbiochem (Cambridge, MA, USA) and the enhanced chemiluminescence (ECL) substrate solution from Bio-Rad Laboratories (Hercules, CA, USA). The radioimmunoprecipitation (RIPA) buffer was purchased from Elpis Biotech. (Daejeon, Korea) and the polyvinylidene fluoride (PVDF) membrane was obtained from Millipore Co. (Billerica, MA, USA). Primary and secondary antibodies were purchased from Bioss Inc. (Beijing, China), Calbiochem Co. (San Diego, CA, USA), Cell Signaling Tech. (Beverly, CA, USA), and Santa Cruz Biotech. (Santa Cruz, CA, USA). Petolinarin (5,7-Dihydroxy 4′,6-dimethoxyflavone 7-rutinoside) was purchased from Sigma-Aldrich (≥98%, HPLC). Pectolinarin was dissolved in DMSO and stored at 4 °C for use, as shown in Figure 5.

### 4.2. OH Scavenging Assay

The method of Gutteridge was used to test pectolinarin’s ·OH scavenging abilities [66]. After mixing 10 mM FeSO_4_·H_2_O-EDTA, 10 mM 2-deoxyribose solution, and the pectolinarin for each concentration, 10 mM H_2_O_2_ was added. The mixture was incubated at 37 °C for 4 h before adding 2.8% TCA and 1.0% TBA solution, boiling at 100 °C for 20 min, ice cooling for 10 min, and measuring absorbance at 490 nm. The formula was as follows:

OH scavenging activity (%) = (Absc − Abss)/Absc × 100

Absc: Absorbance of control, Abss: Absorbance of sample.

### 4.3. NO Radical Scavenging Assay

The method of Marcocci [67] was used to assess pectolinarin’s NO radical scavenging ability. Each pectolinarin concentration was dissolved in methanol, and a 5 mM sodium nitroprusside solution was added and incubated at room temperature for 150 min. After the reaction, the mixed solution was placed in a 96-well plate at a ratio of 1:1 with Griess reagent and reacted at room temperature with light protection for 30 min, and the absorbance was measured at 540 nm. The formula was as follows:

NO radical scavenging activity (%) = (Absc − Abss)/Absc × 100

Absc: Absorbance of control, Abss: Absorbance of sample.

### 4.4. Cell Culture

SH-SY5Y human neuronal cells were cultured in DMEM medium containing 10% FBS and 100 units/mL penicillin–streptomycin in an incubator at 37 °C and 5% CO_2_ humidity. Cells were cultured in a T75 flask, rinsed with phosphate-buffered saline (PBS, PH = 7.4) until they reached a growth state of more than 80% differentiation, and then 2 mL of trypsin-EDTA mixture was added to separate the attached cells before centrifugaton at 1000 rpm for 3 min.

### 4.5. Cell Viability

The MTT technique was used to assess cell viability [68]. When the cells were more than 80% differentiated, 2.5 × 10^5^ cells/well were seeded in a 96-well plate and incubated at 37 °C for 24 h. After 24 h, pectolinarin in different concentrations was added to the plate for each well and cultured for 4 h before being treated with H_2_O_2_ (500 μM) and cultured for another 24 h. After 24 h incubation, the medium was removed from each well, 200 μL of 5 mg/mL MTT solution added, and the plate incubated for 4 h at 37 °C. After that, the MTT solution was removed and DMSO added for 30 min. The absorbance was measured at 540 nm (Thermo Fisher Scientific, Waltham, MA, USA).

### 4.6. Measurement of ROS Production

The DCF-DA assay was used to measure ROS production according to Wang and Zhu’s method [69]. When the cells had been differentiated by more than 80%, they were seeded at 2.5 × 10^5^ cells/well in a black 96-well plate and cultured at 37 °C for 24 h. After 24 h, pectolinarin at different concentrations was added to the plate for each well and cultured for 4 h before being treated with H_2_O_2_ (500 μM) and cultured for 24 h. After 24 h, cells were treated with 80 μM of DCF-DA solution after removing the medium from each well, incubating for 30 min at 37 °C, and then measuring the fluorescence at an excitation of 480 nm and emission of 535 nm using a FLUO starOPTIMA (BMG labtech, Ortenberg, Germany).

### 4.7. Measurement of LDH Release Activity

The LDH release was assessed using an LDH cytotoxicity detection kit, following the manufacturer’s protocol (Takara Bio Inc., Kusatsu, Japan). The cell supernatant was combined with the LDH reaction mixture (catalyst: dye solution = 1:45) and incubated at room temperature for 30 min. The absorbance was measured at 490 nm (Thermo Fisher Scientific, Waltham, MA, USA).

### 4.8. Western Blot Analysis

SH-SY5Y cells were seeded at 1 × 10^6^ cells/mL in a 100 mm cell culture dish, cultured at 37 °C for 24 h, and then treated with pectolinarin (1, 2.5, 5, and 10 μg/mL) and incubated for 4 h before being treated with H_2_O_2_ (500 μM) and cultured for 24 h. After 24 h, the cells were collected and centrifuged at 4 °C for 30 min at 12,000 rpm in RIPA buffer. The supernatant was then transferred to a new e-tube. Each sample was separated by a 10% or 13% sodium dodecyl sulfate-polyacrylamide gel (SDS-PAGE) and transferred to the PVDF membrane by electrophoresis. Blocking was carried out using 5% skimmed milk for 1 h at room temperature, diluting the primary antibody in PBST to a concentration of 1:1000 or 1:200, and reacting overnight at 4 °C. Each of the following primary antibodies was used: β-actin (1:1000; Cell Signaling Tech.); COX-2 (1:500; Calbiochem Co.); iNOS (1:1000; Calbiochem Co.); IL-1β (1:1000; Cell Signaling Tech.); Bax (1:1000; Cell Signaling Tech.); Bcl-2 (1:1000; Abcam, Cambridge, UK); HO-1 (1:2000; Abcam); Nrf2 (1:1000; Cell Signaling Tech.); and Keap1 (1:1000; Cell Signaling Tech.). After washing the membrane, it was reacted with a 1:1000 dilution of the secondary antibody at room temperature for 1 h. The protein expression was confirmed using a Chemiliuminescence image system (Davinch-Chemi^TM^) after washing with 1 × PBST and reacting with ECL solution.

### 4.9. Statistical Analysis

All results are expressed as mean ± standard deviation. Statistical significance was determined using one-way analysis of variance followed by Duncan’s multiple test on the SPSS statistics program (version 25, SPSS Inc., Chicago, IL, USA). Statistical significance was considered as *p* < 0.05.

## 5. Conclusions

In conclusion, pectolinarin had in vitro antioxidant capabilities for ·OH and NO radicals scavenging, increased cell viability, and decreased ROS formation in H_2_O_2_-induced SH-SY5Y cells. In addition, pectolinarin inhibited LDH release in H_2_O_2_-induced SH-SY5Y cells. Furthermore, pectolinarin regulated oxidative stress, inflammation, and apoptosis-related protein expression and provided neuroprotection against H_2_O_2_-induced oxidative stress in SH-SY5Y cells. Therefore, pectolinarin has the potential to be used as a plant material for functional foods, to be applied for the treatment of oxidative stress-related neurodegenerative diseases. In the future, we will investigate the protective mechanisms of pectolinarin against inflammation and oxidative damage in aging mouse brain models, in order to provide a theoretical basis for future research and the development of AD functional foods.

## Figures and Tables

**Table 1:** OH scavenging activity of pectolinarin.

Treatment (μg/mL)	Scavenging Activity (%)
1	63.09 ± 1.97 ^c^
2.5	80.43 ± 1.37 ^b^
5	83.60 ± 0.75 ^a^
10	84.77 ± 0.95 ^a^

Note. Values are mean ± SD. The different letters (a–c) show significantly differences (*p* < 0.05) by the Tukey HSD multiple range test.

**Table 2:** NO radical scavenging activity of pectolinarin.

Treatment (μg/mL)	Scavenging Activity (%)
1	23.21 ± 1.09 ^d^
2.5	24.50 ± 1.37 ^c^
5	30.40 ± 0.59 ^b^
10	35.19 ± 0.32 ^a^

Note. Values are mean ± SD. The different letters (a–d) show significant differences (*p* < 0.05) by the Tukey HSD multiple range test.

## Data Availability

The data presented in this study are available on request from the corresponding author.

## Sample Availabilty

Not available.
